# Supplementary figures and images for: Autoimmunity and clinical pathology amelioration in SLE by dexamethasone primed mesenchymal stem cell derived conditioned media
Source: Stem Cell Res Ther. 2025 Mar 29;16:158. doi: 10.1186/s13287-025-04208-6 (PMC11954324; doi:10.1186/s13287-025-04208-6)

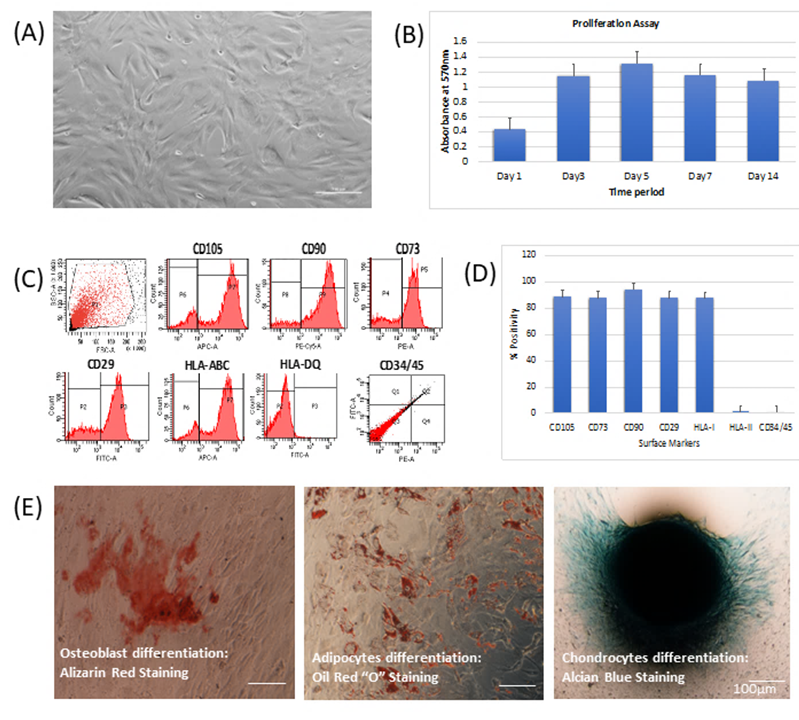

Supplement: Supplementary file 1 — Additional file 1. [file 13287_2025_4208_MOESM1_ESM.tif]
